# Irrespective of the degree of hyperlactatemia, similar lactate levels were associated with a lower mortality rate in metformin users compared with non-users: beware of confounders!

**DOI:** 10.1186/s13613-020-00766-5

**Published:** 2020-10-28

**Authors:** Patrick M. Honore, Leonel Barreto Gutierrez, Luc Kugener, Sebastien Redant, Rachid Attou, Andrea Gallerani, David De Bels

**Affiliations:** ICU Dept., Centre Hospitalier Universitaire Brugmann-Brugmann University Hospital, Place Van Gehuchtenplein, 4, 1020 Brussels, Belgium

We read with great interest the article by Posma et al. who reported that early lactate levels were strongly associated with mortality and, irrespective of the degree of hyperlactatemia, similar lactate levels were associated with a lower mortality rate in metformin (MET) users compared with MET non-users [1]. We would like to make some comments. In their observational study, MET users were more often treated with mechanical ventilation, inotropes or vasopressors, and between 3 and 20% of patients received renal replacement therapy (RRT) [1]. Because of its low molecular weight and minimal protein binding, metformin is equally (highly) eliminated by ultrafiltration (convection) and dialysis (diffusion). Furthermore, its large volume of distribution within a two-compartment pharmacokinetic model implies that metformin may be more effectively cleared by prolonged RRT [2]. This was corroborated by Keller et al. [3] who showed a dramatic reduction of metabolic acidosis and plasma metformin concentrations within the first 24 h after initiating continuous renal replacement therapy (CRRT) in patients with MET-induced lactic acidosis, followed by normalization on the second day in all subjects. The finding that MET users more often require RRT has already been reported in other studies. For instance, in a study by Doenyas-Barak et al. [4], 38.6% of the MET-treated population received RRT, as compared to 21.2% of the cohort of patients not treated with MET. Accordingly, we suspect that the observed difference in mortality rate may be due to the more frequent use of RRT in the MET-treated population. A protective effect of RRT has been suggested by Peters et al. [5] who found that despite higher illness severity, the mortality rate in patients with MET-associated lactic acidosis treated with intermittent hemodialysis was no different to that of non-dialyzed subjects. It would be extremely interesting to know the correction rate of MET and lactate after initiation of RRT in the MET group of Posma et al.

## Data Availability

Not applicable.

## Abbreviations

MET: Metformin
RRT: Renal replacement therapy
CRRT: Continuous renal replacement therapy
